# Community Advisory Board Members Speak Out: Insights From an Opioid Overdose Study

**DOI:** 10.1111/hex.70794

**Published:** 2026-08-02

**Authors:** Deborah Chassler, Ariel Hayes, Craig McClay, Andrea Macone, Magda Colón, Kim Powers, Pedro Alvarez, Andrew Laudate, Gary Langis, Teri Tirado, Tracy Battaglia, Linda Sprague Martinez

**Affiliations:** ^1^ Boston University School of Social Work Boston MA USA; ^2^ Boston University School of Public Health Boston MA USA; ^3^ Boston Medical Center One Boston Medical Center Pl Boston MA USA; ^4^ Yale School of Medicine Yale Cancer Center New Haven CT USA

## Abstract

**Introduction:**

Incorporating the knowledge of people who use/have used drugs into academic research presents the possibility of new ways of understanding drug use, increasing opportunities for equitable outcomes. Community Advisory Boards (CAB) are part of the community‐engaged research continuum offering community members clearly defined roles and a structure to advise academic researchers. Acting in an advisory capacity, CAB members have a platform for sharing community‐informed knowledge with researchers. The concept of knowledge democracy suggests that not all knowledge is valued equally, with scientific knowledge often considered neutral or more valuable than community knowledge which is sometimes understood as subjective opinion. Transformative change requires an embrace of knowledge that extends beyond the sole perspective of academic research to embrace multiple ways of knowing.

**Methods:**

We used directed content analysis to analyze minutes from 43 monthly CAB meetings, focusing on member knowledge about opioid use and overdose. We centre the expertise of people who use/have used drugs and propose that academic researchers should listen to and embrace knowledge that is held by the communities and people being studied. Aims: (1) identify and describe the knowledge Massachusetts CAB members shared with academic researchers and each other during a federally‐funded study to reduce opioid overdose fatalities and (2) centre the contributions of community knowledge for the study.

**Results:**

Three themes were identified: (1) CAB members generously shared their knowledge; (2) Members brought attention to root causes of opioid overdose fatalities; and (3) Honest and challenging meeting discussions created solidarity and trust among members and with staff.

**Conclusions:**

This case study makes an important contribution to the literature by highlighting the knowledge that a Community Advisory Board, including people with lived and living experience of drug use, can bring to substance use research. By centreing the voices of people with lived and living experience, front line staff, and family members, the study confirms that a substance use research‐focused CAB can be an important source of valuable knowledge, contributing to the development of knowledge democracy.

**Public Contribution:**

The HEALing Communities Study Massachusetts Community Advisory Board members ‐ people with lived and living experience with their own or family member drug use, harm reductionists, recovery coaches, and public health advocates committed to saving the lives of people who use drugs – were active participants in the HEALING Communities Study. They were involved in local, statewide and national levels of the study, including community coalitions, study cores, the national steering committee, and dissemination, acting as advisors and consultants with staff throughout the 4 years of the study and beyond. Eighteen months after the study officially ended, members continue to stay in frequent contact with each other and staff and many chose to be co‐authors of this manuscript.

## 1 Introduction

The urgency of challenging politically palatable responses to address loss of life from the opioid overdose epidemic cannot be understated. Incorporating the knowledge of people who use or who have used drugs into academic research is essential for reducing overdose deaths and increasing opportunities for equitable outcomes. People with lived or current experience of drug use understand the context of community needs [1] and have developed a number of highly regarded practices for saving lives, e.g., syringe exchange programs [2]. Community‐engaged methods, including Community Advisory Boards (CAB), are widely used by academic researchers. However, few CABs have engaged people who use drugs, their families, and direct service providers, to reduce opioid overdose deaths.

The federally‐funded four‐state (Kentucky, Massachusetts, New York, Ohio) HEALing Communities Study (HCS) aimed to reduce opioid overdose fatalities using a community‐engaged coalition‐driven randomised‐cluster waitlist control trial which engaged community members, including people who use/used drugs, their family members, providers, and friends and neighbours [3]. Each state was asked to establish a CAB to advise the study on its goal to reduce opioid overdose fatalities.

The knowledge of people from stigmatised or marginalised communities can be perceived as less valuable than scientific knowledge, ‘relegated by modern science to the realm of beliefs, opinions and intuitive or subjective understandings…’ [4], (p. 12). In a partnership between CAB members and academic researchers to reduce opioid overdose fatalities and address existing inequities in fatality rates, both community members and academic researchers need to be aware of their assumptions about what they understand to be valuable knowledge [5, 6]. According to Hall and Tandon [5] ‘knowledge democracy’ decries the enclosure of knowledge in the academy and encourages engaging a bidirectional flow of knowledge from marginalised communities to the academy. Mullett [7] suggests that to succeed in fostering knowledge democracy, researchers should be willing to change, including developing new kinds of research relationships. Gilbert and colleagues (2023) suggest that transformative change requires us to embrace multiple ways of knowing and collaborative partnerships.

Community Advisory Boards (CAB) are part of the community‐engaged research continuum offering community members clearly defined roles and a structure to advise academic researchers [8, 9, 10]. As such, a CAB provides a platform for sharing community‐informed knowledge with researchers [11]. Farhang and Morales [1] suggest that community‐engaged research should centre the expertise of community members [12]. Pursley and colleagues found that members appreciate a mission they can commit to, a focus on community needs, and working together with respect. HCS CAB meetings were structured to build capacity for knowledge sharing and appreciation of diverse perspectives among CAB members and research staff [12].

### 1.1 Purpose

The HCS MA CAB convened 43 times over the 4 years of the study generating a treasure trove of monthly meeting minutes which we used for this case study. Case study aims include: (1) identify and describe the knowledge Massachusetts CAB members shared with academic researchers and each other during monthly meetings of a federally‐funded study to reduce opioid overdose fatalities and (2) centre the contributions of community knowledge to the study. In this manuscript, we centre CAB knowledge about opioid overdose fatalities, addressing a gap in the literature. We highlight member willingness to share their knowledge and challenge each other and staff. Advarra Inc., served as the HEALing Communities Study Single Institutional Review Board. This study protocol (Pro00038088) had no human subjects. Study staff performed study activities complying with relevant laws and institutional guidelines which were approved by the appropriate institutional committee(s).

## 2 Methods

### 2.1 Setting and Participant Recruitment

Eight at‐large Massachusetts HCS CAB members with statewide knowledge and experience advised on the development of the HCS proposal. Once funded, one at‐large member became staff. The remaining 7 at‐large members recruited and selected 16 community members from participating municipalities to represent the 16 study communities which were randomised into an intervention group (Wave 1) and waitlist control group (Wave 2). Recruitment and selection focused on increasing the diversity of the CAB to include a range of identities, work sectors, and roles in the community, and not least, to include people with a history of or current drug use experience (For more background see [13, 14, 15, 16]. CAB recruiting materials asked about the applicant's experience using drugs; age; identities including gender, race, sexual orientation, veteran status, immigrant, user of SUD services, housed or experiencing homelessness, and incarceration experience, and; professional affiliation, if any.

### 2.2 Design of the HCS CAB

Beginning in February 2020 and ending in December 2023, the 7 at‐large members and 16 community CAB members (*n* = 23) and study staff (averaging 5–10 staff) including the Principal Investigator and Project Director, attended 38 2‐h monthly Zoom meetings and five all‐day in‐person meetings. During the Covid‐19 epidemic, meetings were virtual until May 2022, when we added twice‐yearly full‐day in‐person meetings to the monthly Zoom meetings. Designated study staff took detailed written minutes at all 43 meetings, with multiple notetakers at in‐person meetings. Minutes contained verbatim quotes from CAB members and staff as well as brief summaries of what was said if the person taking the minutes could not capture the exact quote. Meetings were not recorded. Minutes were reviewed first by the CAB facilitator, and then sent to all meeting attendees each month, for review and acceptance. For this manuscript, all quotes are from CAB members; their names and communities were removed.

### 2.3 Participation

At the start of the study in 2020, the CAB had 7 at‐large members and 16 community members (*n* = 23). A total of 12 members remained on the CAB for all 4 years including 10 of the original 16 community members. To ensure that we had representatives from all 16 communities, new members were recruited and selected to replace any community member who stepped down. All CAB members could opt‐in to receive compensation for meeting attendance, as well as travel costs and time spent travelling for in‐person meetings.

### 2.4 Data and Data Analysis

For this case study we focused specifically on the HCS Massachusetts CAB which all authors, including Massachusetts CAB members and study staff who attended CAB meetings, knew well. We did this because detailed Massachusetts CAB meeting minutes were readily available; meetings had good attendance from members and staff; staff were intentional in their community‐engagement practices; the Principal Investigator and Project Director attended nearly every meeting demonstrating their commitment to the CAB members; the CAB met monthly despite Covid‐19 (on zoom for the vast majority of the meetings), and; Massachusetts members and staff were explicitly committed to forming a CAB that included members with lived or living experience.

All meeting minutes were saved in NVivo 14 [17]. With CAB and community engagement literature as the foundation, we used directed content analysis to code the minutes [18]. Two coders, the first and second authors, reviewed the first 6 months of meeting minutes and created a codebook with over 20 codes and subcodes. Next, we continued coding all 43 months of meeting minutes, aligning codes as needed. For this case study about CAB member knowledge, we used four codes: (1) knowledge about stigma related to drug use/drug treatment; (2) member challenges and suggestions directed to study staff; (3) engagement in mutual learning and growing; and (4) member interactions with each other and staff. These codes describe specific aspects of CAB member statements during meetings.

We developed coding summaries for the four codes and moved to developing themes [18, 19]. Each coder kept a journal of memos, annotations and reflections and discussed these with each other at weekly meetings. The first theme ‘CAB members generously shared their knowledge’ developed through our reflections on the different ways CAB members expressed their knowledge and introduced mutual learning to meetings, the first and third codes. Mutual learning (the third code) informed the second theme ‘Members brought attention to root causes of opioid overdose fatalities’ which also described member challenges to study staff to recognise the root causes leading to overdose fatalities (the second code). The third theme, ‘Honest and challenging meeting discussions created solidarity and trust among members and with staff,’ brings together ideas from the mutual learning code and members' interactions with each other and staff, the fourth code.

### 2.5 Reflexivity

The first two authors used reflexivity exercises outlined by Olmos‐Vega and colleagues [20]. We are both White identified and aware of our positions in academia, a senior researcher and a younger graduate student, and noted our standpoints and power. We both have personal and work experience relevant to drug use and acknowledge how that affects how we understand the words of CAB members. We share racial and social justice values that influenced our perspectives on topics the CAB members raised. With awareness of our standpoints, we stayed close to the content of the minutes, allowing member statements to be the focus of the study. The first author was present at every CAB meeting and intentionally developed ongoing relationships with CAB members that continue to this day. The second author was a graduate student who had no prior experience with the HCS MA CAB or the minutes.

#### 2.5.1 Personal Reflections on the Role of CAB Facilitator

As the CAB facilitator, my goal was to create a meeting space open to multiple points of view, one with room to interrogate assumptions about drug use and people who use drugs; recovery and harm reduction; and the accepted academic science of drug addiction and treatment. When facilitating the CAB meetings, I helped members and staff stay with conversations that felt uncomfortable, thanking people for their contributions, allowing for silence when needed, and creating space for questions, comments, and rebuttal. Each month I called, texted, or emailed each CAB member, getting to know them and learn about their interests, questions, frustrations and suggestions. While I worked with staff to make sure they were getting the advice and information from the CAB that they wanted, I frequently followed the lead of members.

Each CAB meeting had a carefully designed agenda, usually the result of collaborations among the facilitator, study staff, and members. Meetings provided a platform to share knowledge, challenge assumptions, and discuss concerns, creating a space honouring multiple perspectives and different knowledge about drug use, recovery, treatment, and harm reduction.

## 3 Results

Using summaries of the four codes about CAB member knowledge, we identified three themes: (1) CAB members generously shared their knowledge; (2) Members brought attention to root causes of opioid overdose fatalities; and (3) Honest and challenging meeting discussions created solidarity and trust among members and with staff.

### 3.1 CAB Members Generously Shared Their Knowledge

CAB members asserted that people with lived experience could offer practical strategies to reduce overdose fatalities. In the first years of the study some CAB members questioned whether the study was truly committed to integrating perspectives of people with lived experience into study activities.

[Member] said that lived experience brings in the most recent and current information about the landscape, impact, etc.

[Member said I] Understand the study is focusing on evidence‐based practices but frustrated that [study] coalitions cannot do interventions more outside of the box….

Some members expressed deep concern and sometimes frank frustration that people who used drugs were not valued for their contributions of wisdom and experience. Members said that it is not enough to have a seat at the table; people who use drugs should be included in decisions, noting the benefits that would accrue through doing so.

[Member] shared the following reflection in the chat: As always we have to prove our value. The end game is to make them see we do things better and moving forward we can take over the care. They have too much power still, but we have to work at chipping it away and giving it back to ourselves.

While the study focused on providing medications for opioid use disorder, members advocated for first building relationships with people who are using drugs.

[Member] you can meet people in encampments. She said that it takes a while to make these connections, but to look for low‐income housing, talk to police departments and ask them where are people using, where are people overdosing. When you get in there and get trust with one person you can spark a chain of connections.

Members wanted people who used drugs to be integral to the study because, as they described, the knowledge that people with lived experience could offer to the study is valuable. They pointed out that people are continuing to die from overdoses, suggesting that what was being done by the study was not yet bringing down overdose rates, most especially among communities of colour.

We're not focusing on the individuals that are still out there struggling.

Members questioned how study funds were being distributed and emphasised the urgency of doing things differently.

[CAB members expressed] Frustration around some positions that can't be funded. CAB wants to give money to people who are doing overdose prevention on the street and not stick to [study] HCS notions of who is ‘qualified’ to do this work…. This would include changing the job descriptions so that applicants are not required to have a phone or car to get the job.

There is a lot of programmatic perspective at the table, but in practice, when the rubber meets the road, the perspective of those being impacted is missed.

Members understood that people who are supporting study interventions, working with people who use drugs, do not get paid enough, and they wanted to do something about that.

It would be helpful to have more autonomy on pay and how much we pay people.

Sometimes the worst thing that can happen is when the government thinks that they have the best way to spend the money.

Members created a list describing the ways members and staff could hold themselves accountable for saving lives.

− Holding accountable organisations that have power and money
− Assisting people to navigate the system
− Person‐centred approach
− No lip service, keep in mind the grant is for those actively using

Members repeatedly reminded each other and study staff to value people who use drugs, noting that we sometimes value some people more than others.

…we have to be very careful not to set up a hierarchy of who is more valuable. We should not be saying, for example, a sober individual is more important or valuable than someone currently using.

Those in power keep trying to get ‘those people' off the street, to hide people away, pretending they don't exist and don't need help.

Members called attention to the importance of including people who use drugs in decisions that will impact them, offering examples of ways their perspectives were, perhaps, not taken into consideration.

[Member said] that he means no disrespect to the work that the recovery center is doing, but there are not a lot of spots for people with an active addiction to go to.

If I go to get a job and they do a drug test and it comes back positive, I don't get the job – this is excluding people; need to have a system for people who are actively using.

### 3.2 Members Brought Attention to Root Causes of Opioid Overdose Fatalities

CAB members discussed the root causes of the opioid epidemic including stigma, racism, criminalisation of drugs, identified the impact of root causes on their communities and the state, and engaged in collective group learning sessions drawing on the unique expertise of each person. Members grappled with structural and systemic racism and drew attention to the way racism harms people of colour who use drugs. They emphasised that criminalisation of drug use and punitive approaches to drug treatment are harmful and described harm reduction approaches which keep people alive.

We can't reduce opioid death by 40% without addressing White supremacy and social determinants of health.

Police, treatment facilities, hospitals, health centres all have deep systemic racism that determines who receives care and treatment.

…police approaches [to helping people who use drugs] might not be entirely appropriate as Black and Hispanic populations tend not to want to work with the police.

…conversations about equity are not comfortable but need to be had. People need to understand these conversations are not a personal attack or accusation, and you need to be allies when you show up…Racism is not an accusation; it is a fact. Put skin in the game. How do you show up?

Members pointed to the need for resources and services to be available in multiple languages, and to ‘make sure everyone has as many resources as White people have.’

A lot of pharmacies that distribute buprenorphine are in predominantly White areas….

…it is all too easy for communities to say that most of their community is White and ignore other demographics.

Syringe exchanges have historically been set up for White people. When you step outside the brick and mortar and enter the community, you see people from the community [including people of colour], but inside [the syringe exchange] you are not always seeing the people from the community …. [Member] gets tired of explaining it, there is always push back of getting [syringe] programs approved in inner cities…

Members noted that services often do not reach the people most harmed by opioid overdose, sometimes because providers are not aware of the way communities differ from one another.

…do not assume IDU [injection drug use] as a default in your community, or among different groups in the community.

…it is important to know the culture of your community.

…it needs to be up to the community and they need to give their input. What works in [one community], may not work in [another community].

A CAB member explained the mismatch between the utility of a mobile van and the needs of people who are using drugs.

As someone who injected drugs, I wasn't walking around from 8 to 5. [Member] raised the point that if these vans [mobile vans] are in parking lots during these hours then they won't be effective if people are not out until after dark. She said that we need to be meeting people using drugs, who are not accessing harm reduction facilities, and who are not going to the services and [we need to be] working with the people who have connection to these folks.

CAB members grounded their insights into solutions and strategies that keep people alive.

[Member] reminded us that people don't die if they are supervised while using drugs.

Members described pervasive stigma towards people who use drugs, including in healthcare systems. Members also described stigma among family members, in hospitals and among service providers including doctors, nurses, pharmacists, recovery coaches and among people who use drugs (internalised stigma).

[Because of stigma] people are reluctant to go to the hospital, and they could die because they refuse to go to the hospital for treatment.

[Member] said that people will give up on other people so easily, because they'll say this person doesn't want to get sober…

A community agency participating in the study used a harm reduction approach to address stigma, providing financial supports for peers, demonstrating respect and creating trust with the community.

[CAB member said] this question about paying peers comes from a place of stigma…. When people have choices and self‐determination, and there is no coercion, all of a sudden they support each other. If we help people support themselves, they will start supporting others and develop their own community. Those are the champions to pay.

As one CAB member said, ‘Stigma is what ends up killing people. Everyone should have access to services.’

### 3.3 Honest and Challenging Meeting Discussions Created Solidarity and Trust Among Members and With Staff

CAB members affirmed the values and expertise they brought to the study and encouraged each other to continue to persevere. They challenged commonly held perspectives on drug use, people who use drugs, recovery and harm reduction.

We/the CAB are not the voice of science, research, documentation or statistics. The CAB is the VOICE of the streets, of those suffering and their families, of what works and doesn't work, of what folks in active use will and won't do.

Members sometimes disagreed with each other. They also appreciated one another's thoughtful critiques. Members acknowledged listening, contributing, and learning from each other.

[Member] learned about himself, that at the beginning, his understanding of MOUD and harm reduction was very narrow. But with more education, his perspective began to widen with all the information he learned.

[Member] and [another member] both shared how the CAB makes them feel valued, and love being able to contribute and hear different perspectives.

Members educated each other and staff about harm reduction and recovery. Several members identified as ‘being in recovery' while others questioned the concept of ‘recovery,' suggesting that it was neither specific nor inclusive enough to capture the lives and experiences of people who have used and may continue to use drugs.

Members focused on changing treatment policy to make services available wherever people are using drugs rather than relying on them to come into an office to receive services.

They have started outreach at housing locations in [community] doing food distribution and naloxone training. Overdoses have decreased and naloxone distribution has increased.

One member had a larger vision of what access to services could look like, including giving people access to both recovery and harm reduction services at the same time in the same place. He recommended that staff should be trained to understand and provide both.

… we need people in these [recovery and harm reduction] spaces, like the CAB members, to infiltrate the recovery centres and promote harm reduction. It is hard to keep things as separate entities in [our community] because people are accessing both services. We need to have these connections with people. He said that the premise is around educating the providers……[We need] not just a safe consumption site. [We need a] one stop place for low‐barrier access to all types of services (job training, showers, laundry, wifi, safe place to hang out). Comfortable space for people who use drugs and that is stigma‐free.

At the first CAB meeting, CAB members emphasised collaboration and accountability.

Strong collaboration, value all perspectives. Be explicit in outlining accountability related to equity.

In other meetings they told each other how important it is to speak up.

Make sure you have a voice, make your voice loud, use that voice often and everywhere.

[Member] said to not be afraid to push back on things, speak up and advocate on behalf of the people that you work with.

Members shared information and affirmed each other's contributions. Affirmations cited the strengths each member contributed, describing what they bring to the study is unique and important.

Needs to be super emphasised that the CAB doesn't bring a clinical, academic, data voice – the CAB brings a voice that no one else can bring.

I so appreciate CAB members speaking their truth and so powerfully.

For HCS MA CAB members, the issue of opioid overdose deaths was profoundly personal. Many members, if not most, had lost people they loved, had lived or living experience of substance use and worked in substance use recovery and harm reduction. At the end of 4 years members described feeling heard and supported by staff [13]. CAB members sometimes disagreed with each other, yet were open to discussion, learning, and growing. They trusted each other, asked questions, and refined their perspectives as they talked. Many points were made repeatedly with patience and persistence. These include: respect and love everyone, including people who are using drugs or have used drugs; value and honour what you learn from people who use drugs; acknowledge the underlying root causes of racism and stigma that have created unjust systems and structures that punish and incarcerate; build solidarity with collaborative learning, acknowledging each other's knowledge, earning each other's trust. Practice harm reduction.

## 4 Discussion

To our knowledge, this is the first case study to leverage written CAB meeting minutes from an opioid overdose reduction research study to systemically describe the unique knowledge and contributions of community members with lived or living experience of drug use, family members, direct service providers and friends and neighbours. With this case study, we extend the CAB literature to focus on knowledge CAB members shared over a 4‐year research effort to reduce opioid overdose fatalities in 16 communities in Massachusetts. CAB meeting minutes were used to identify, describe and centre CAB member knowledge shared during 43 meetings. Doing so highlights knowledge democracy, disrupting beliefs that socially‐credentialed perspectives must be placed within and at the top of a value hierarchy.

A rich substance use research literature that includes people with lived and living experience demonstrates the value of community‐based knowledge and its impact. Kim [21] asserts that ‘experiential knowledge’ can challenge and change research protocols. Greer and colleagues [22] and Jalloh and colleagues [23] emphasise that engaging ‘peers’ improves substance use research. Community members often highlight harm reduction principles and practices, the type of care offered, and when and how it is offered (See, e.g. [24, 25, 26]). Insights from people who use drugs and engage with health care are critical for addressing needed health care systems change [27, 28, 29, 30]. And, as the title of a recent article explains, the reason to centre the knowledge of people who use drugs is ‘Because we're the ‘experts’ [27].

This case study reflects the research described above. CAB members used their experiences and knowledge about drug use to advise the study. They were steadfast advocates for the integration of people with lived and living experience of drug use into the study at every level. They recognised their own expertise, repeatedly reminding each other and staff that people who have used drugs must contribute to the work of reducing overdose deaths [2, 31, 32]. They focused on community and individual needs as defined by those with lived and living experience. They described how existing policies and medical solutions developed by academic research were not fully addressing the multiple needs of people using drugs. CAB members' awareness of stigma and racism as root causes of opioid overdose fatalities grounded their insights into solutions and strategies to keep people alive. They used meeting time to hold themselves and staff accountable for saving *all* lives. In doing so, they often challenged existing narratives about how best to reduce opioid overdose fatalities.

The HCS MA CAB meetings were designed to value everyone's knowledge. Using small groups to maximise member/staff interactions was one way to nurture mutual learning and respect and strengthen the community/academic partnership. Study staff and CAB members listened, questioned, and learned from each other. Over the course of the study, members shared community victories and what it took to arrive there, shared strategies for engaging underserved communities of colour and shared their personal journeys. Members and staff discussed future funding opportunities, resources, and professional development opportunities.

Boundary spanners are people in organisations who bridge the boundaries which demarcate individual roles, knowledge, and power, thereby enhancing collaboration and partnership [33]. Some CAB members bridged the boundaries between staff and members, helping each to work together in partnership during meetings, in individual conversations, and in the active roles they played across the study. Boundary spanners also play an important role in the development of mutual trust. CAB member boundary spanners helped other members and staff to value new knowledge, leading to increased knowledge democracy.

The current case study advances knowledge democracy by using HCS MA CAB meeting minutes to document and reflect on CAB members' knowledge about opioid overdose fatalities in their communities and across the state. Because the minutes contain a substantial amount of rich data, we made an analytic decision to write separate case studies that highlight different aspects of the CAB process. The parent HCS study was designed as a community‐engaged, community‐driven initiative grounded on the premise that community members hold essential expertise for informing solutions to reduce opioid overdose fatalities. The creation of a statewide CAB was itself an acknowledgment of the value of learning from CAB members.

An earlier case study used the same meeting minutes to examine staff community engagement practices, including how staff responded to and acknowledged CAB member knowledge [13]. In contrast, the present case study intentionally centres CAB member statements and the knowledge and advice they shared with each other and with staff, without analysing staff responses or changes in staff practice. This analytic choice allowed us to foreground CAB expertise, avoiding conflating it with staff interpretation or action. Centreing CAB knowledge without simultaneously analysing staff responses also allowed us to avoid tokenizing CAB contributions, treating member expertise as worthy of standalone examination.

The absence of explicit documentation of staff learning or changes in the research design represents a limitation of this case study. Because staff reflections and adaptations were not systematically captured in the minutes used for this analysis, this study cannot speak to the impact of CAB contributions on staff or on the broader study. Future analyses could re‐examine the minutes with this specific focus or incorporate additional data sources to assess how staff learning occurred and how CAB input shaped the study over time.

In its advisory capacity, a CAB often has little power to decide on study design, interventions, and policy [34, 35], limiting opportunities to utilise the power of CAB knowledge. While the premise of the HEALing Communities Study was that community knowledge could help researchers reduce opioid overdose fatalities, the CAB was limited to advising on the research [36]. Critically, both CAB members and HCS staff demonstrated commitment to the CAB: HCS invested time and money in the development of the CAB with members attending well‐compensated monthly meetings for the 4 years of the study; staff regularly attending CAB meeting, and; inclusion of members across the study [13]. CAB members celebrated the opportunity to contribute their knowledge and transform how best to address opioid overdose fatalities. In doing so, they formed lasting relationships with each other and with staff and strengthened knowledge democracy.

## 5 Conclusion and Future Directions

This study suggests that a substance use research Community Advisory Board which centres the voices of people with lived and living experience, front line staff, and family members, is an important source of valuable knowledge. CAB members, deeply connected to the communities of people who use drugs, shared the power of their knowledge and their commitment to saving lives.

Knowledge democracy asserts that we each possess valuable knowledge. Critical examination of knowledge can move us towards justice and equity for all [5]. CAB members used their knowledge to educate each other and staff about ways to save lives, promote equity and justice, decrease stigma and increase access to services that would help save the lives of people most at risk for overdose. They did not hold back. They held themselves accountable for reducing fatal overdoses and they made sure they had a say in creating the future for people who use drugs. Members stayed in touch with each other, knew that the work was not yet finished, and said yes to opportunities to continue the work.

Next steps for increasing knowledge democracy include collaborations between academic researchers and community members to co‐design research projects and share decision‐making power and resources to create innovative, equitable and justice‐focused practices and policies. Future studies should build upon foundational work that centres community knowledge while engaging in comparative CAB studies or a longitudinal exploration of CAB impact on substance use research studies. The literature would also benefit from further research to operationalise community knowledge in research and policy contexts while continuing to disrupt traditional power dynamics.

## Author Contributions

**Deborah Chassler:** investigation, writing – original draft, methodology, writing – review and editing, formal analysis, supervision, project administration. **Ariel Hayes:** writing – original draft, writing – review and editing, formal analysis, investigation, methodology. **Craig McClay:** writing – review and editing. **Andrea Macone:** writing – review and editing. **Magda Colón:** writing – review and editing. **Kim Powers:** writing – review and editing. **Pedro Alvarez:** writing – review and editing. **Andrew Laudate:** writing – review and editing. **Gary Langis:** writing – review and editing. **Teri Tirado:** writing – review and editing. **Tracy Battaglia:** writing – review and editing, funding acquisition. **Linda Sprague Martinez:** conceptualisation, writing – review and editing, funding acquisition, methodology.

## Ethics Statement

All procedures were performed in compliance with relevant laws and institutional guidelines and have been approved by the appropriate institutional committee(s). This study protocol (Pro00038088), which has no human subjects, was approved by Advarra Inc., the HEALing Communities Study Single Institutional Review Board.

## Conflicts of Interest

Deborah Chassler reports financial support from the National Institute on Drug Abuse (NIDA) and the Substance Abuse and Mental Health Services Administration (SAMHSA). Linda Sprague Martinez, Tracy Battaglia, Ariel Hayes, Craig McClay, Andrea Macone, Gary Langis, Andrew Laudate, Kim Powers, Pedro Alvarez, Teri Tirado and Magda Colón declare that they have no known competing financial interests or personal relationships that could have appeared to influence the work reported in this paper.

## Data Availability

The parent study, the NIH HEAL Initiative, was a federally funded four‐state research study, each with a Community Advisory Board (CAB). Our research used 43 months of Massachusetts Community Advisory Board CAB meeting minutes to describe the development of the CAB. No CAB member, community, staff or national leadershipnames are associated with quotes in this publication. We are unable to make the meeting minutes available because they are not de‐identified.

## References

[1] L. Farhang and X. Morales , “Building Community Power to Achieve Health and Racial Equity: Principles to Guide Transformative Partnerships With Local Communities,” NAM Perspectives 2022 (June 2022): 1–5, 10.31478/202206d. PMC9499374 36177209

[2] Z. R. Salazar , L. Vincent , M. C. Figgatt , M. K. Gilbert , and N. Dasgupta , “Research Led by People Who Use Drugs: Centering the Expertise of Lived Experience,” Substance Abuse Treatment, Prevention, and Policy 16, no. 1 (September 2021): 70, 10.1186/s13011-021-00406-6. 34544478 PMC8454046

[3] HEALing Communities Study Consortium , J. H. Samet , N. El‐Bassel , et al., “Community‐Based Cluster‐Randomized Trial to Reduce Opioid Overdose Deaths,” New England Journal of Medicine 391, no. 11 (2024): 989–1001, 10.1056/NEJMoa2401177. 38884347 PMC11761538

[4] C. Adelle , “Creating Knowledge Democracy in South Africa: The Role of Communities of Practice,” South African Journal of Science 115, no. 7/8 (2019): 1–3, 10.17159/sajs.2019/5888.

[5] B. Hall and R. Tandon , “Decolonization of Knowledge, Epistemicide, Participatory Research and Higher Education,” Research for All 1, no. 1 (2017): 6–19, 10.18546/RFA.01.1.02.

[6] C. T. Yan , Y. Jin , E. Chalfin , and L. Sprague Martinez , “Interrogation, Negotiation, and Subversion of Power Differentials in Community‐Based Participatory Research: A Scoping Review,” Progress in Community Health Partnerships 18, no. 2 (2024): 295–305. 38946574

[7] J. Mullett (2015). Issues of Equity and Empowerment in Knowledge Democracy: Three Community Based Research Examples. Vol. 13, 248–361, 10.1177/1476750315573590.

[8] J. R. Halladay , K. E. Donahue , B. Sleath , et al., “Community Advisory Boards Guiding Engaged Research Efforts Within a Clinical Translational Sciences Award: Key Contextual Factors Explored,” Progress in Community Health Partnerships 11, no. 4 (2017): 367–377, 10.1353/cpr.2017.0044. 29332850 PMC8585511

[9] S. D. Newman , J. O. Andrews , G. S. Magwood , C. Jenkins , M. J. Cox , and D. C. Williamson , “Community Advisory Boards in Community‐Based Participatory Research: A Synthesis of Best Processes,” Preventing Chronic Disease 8, no. 3 (2011): A70. 21477510 PMC3103575

[10] N. P. Yuan , B. M. Mayer , L. Joshweseoma , D. Clichee , and N. I. Teufel‐Shone , “Development of Guidelines to Improve the Effectiveness of Community Advisory Boards in Health Research,” Progress in Community Health Partnerships 14, no. 2 (2020): 259–269, 10.1353/cpr.2020.0026. 33416647 PMC8530020

[11] M. R. Isler , M. S. Miles , B. Banks , et al., “Across the Miles: Process and Impacts of Collaboration With a Rural Community Advisory Board in Hiv Research,” Progress in Community Health Partnerships 9, no. 1 (2015): 41–48, 10.1353/cpr.2015.0014. 25981423

[12] H. Pursley , E. M. Staab , A. Mayer , et al., “Partnership in Promoting Community Health Research: Ten‐Year Evaluation of the Little Village Community Advisory Board,” Progress in Community Health Partnerships 16, no. 4 (2022): 503–515, 10.1353/cpr.2022.0072. 36533500

[13] D. Chassler , A. Hayes , C. McClay , et al., “Stories From Four Years of Monthly Community Advisory Board (Cab) Meeting Minutes: Staff Community Engagement Practices in the Co‐Development of a CAB,” Journal of Community Practice (In Press).

[14] J. Bosak , M.‐L. Drainoni , M.‐C. Christopher , et al., “Community Advisory Board Members' Perspectives on Their Contributions to a Large Multistate Cluster RCT: A Mixed Methods Study,” Journal of Clinical and Translational Science 8, no. 1 (2023): e1, 10.1017/cts.2023.673. 38384918 PMC10879854

[15] D. Chassler , D. McClay , M. D'Onfro , et al., “Work Really Is Being Done and It's Very Worthwhile": Reflections From Community Advisory Board Members,” Progress in Community Health Partnerships 19, no. 2 (2025): 219–226, 10.1353/cpr.2025.a965359. 40718919 PMC12703719

[16] G. A. Hamilton , L. Sprague Martinez , J. A. Barocas , et al., “The Process and Cost of Developing a Community Advisory Board Focused on Opioid Overdose Deaths,” Progress in Community Health Partnerships: Research, Education, and Action 19, no. 3 (2025): 335–343, 10.1353/cpr.2025.a970161. 41017498 PMC12758374

[17] Lumivero , (2025), NVivo (Version 14) [Computer software], https://lumivero.com/products/nvivo/.

[18] H. Hsieh and S. Shannon , “Three Approaches to Qualitative Content Analysis,” Qualitative Health Research 15, no. 9 (2005): 1277–1288, 10.1177/1049732305276687. 16204405

[19] M. Vaismoradi , H. Turunen , and T. Bondas , “Content Analysis and Thematic Analysis: Implications for Conducting a Qualitative Descriptive Study,” Nursing & Health Sciences 15, no. 3 (2013): 398–405, 10.1111/nhs.12048. 23480423

[20] F. M. Olmos‐Vega , R. E. Stalmeijer , L. Varpio , and R. Kahlke , “A Practical Guide to Reflexivity in Qualitative Research: Amee Guide No. 149,” Medical Teacher 45, no. 3 (2023): 241–251, 10.1080/0142159X.2022.2057287. 35389310

[21] C. Kim , “The Engagement of People With Lived Experiences in Substance Use Research,” Frontiers in Public Health 13 (2025): 1611836, 10.3389/fpubh.2025.1611836. 41103477 PMC12521110

[22] A. M. Greer , A. Amlani , B. Pauly , C. Burmeister , and J. A. Buxton , BMC Public Health 18 (2018): 834, 10.1186/s12889-018-5765-2. 29976169 PMC6034321

[23] C. Jalloh , S. Illsley , J. Wylie , et al., “What Goes Around: The Process of Building a Community‐Based Harm Reduction Research Project,” Harm Reduction Journal 14 (2017): 73, 10.1186/s12954-017-0199-1. 29145882 PMC5689138

[24] H. Lokko and W. M. K. Avor , “On the Pathway to Health Equity: Creating a Harm Reduction Strategy for a Large Academic Acute Care Hospital,” Healthcare Management Forum 38, no. 1 (2024): 23–29, 10.1177/08404704241264243. 39037245

[25] L. M. Boucher , Z. Marshall , A. Martin , et al., “Expanding Conceptualizations of Harm Reduction: Results From a Qualitative Community‐Based Participatory Research Study With People Who Inject Drugs,” Harm Reduction Journal 14 (2017): 18, 10.1186/s12954-017-0145-2. 28494774 PMC5427533

[26] K. Dunham , C. Rivas , P. Medina Blanco , et al., “It's Like A Partnership”: Exploring the Primary Care Experiences and Patient‐Defined Goals of People Who Use Drugs,” Journal of General Internal Medicine 39, no. 9 (2024): 1681–1689, 10.1007/s11606-024-08743-5. 38578536 PMC11255174

[27] E. J. Bailey , M. Becker , J. Morrow , et al., “Because We're the Experts': Centering the Expertise of People Who Use Drugs to Identify and Propose Solutions for Overdose Prevention,” Harm Reduction Journal 23 (2026): 14, 10.1186/s12954-025-01368-9. PMC12849431 41454367

[28] R. McBeth , C. Sandberg , V. Varewny , et al., “Co‐Creating Health System Innovation With People Who Use Drugs,” Harm Reduction Journal 22 (2025): 176, 10.1186/s12954-025-01326-5. 41126202 PMC12542041

[29] T. Wideman and S. Karsten , Walk With Me Team ., “Walk With Me: Reducing Harm and Confronting the Toxic Drug Poisoning Crisis in Small British Columbia Cities Through Community Engaged Research,” Harm Reduction Journal 21, no. 1 (2024): 106, 10.1186/s12954-024-01022-w. 38822343 PMC11140918

[30] G. Brown , S. Crawford , G.‐E. Perry , et al., “Achieving Meaningful Participation of People Who Use Drugs and Their Peer Organizations in a Strategic Research Partnership,” Harm Reduction Journal 16 (2019): 37, 10.1186/s12954-019-0306-6. 31182099 PMC6558880

[31] M. M. Medlock , R. Schottenfeld , M. Abdul‐Salaam , et al., “The Community Is the Cure: How African American Washington, Dc Residents Informed Opioid Treatment Engagement,” Progress in Community Health Partnerships 18, no. 2 (2024): 235–245, 10.1353/cpr.2024.a930719. 38946568

[32] S. Byiringiro , G. C. Bellinger , A. Mezu , et al., “From Dialogue to Action: Community Recommendations for Inclusive Research Participation Among Underrepresented Populations,” Health Expectations 28, no. 4 (2025): e70348, 10.1111/hex.70348. 40739722 PMC12310556

[33] M. Hatch , J. Parrish , S. Heppell , et al., “Boundary Spanners: A Critical Role for Enduring Collaborations Between Indigenous Communities and Mainstream Scientists,” Ecology and Society 28, no. 1, Article (2023): art41, 10.5751/ES-13887-280141.

[34] K. L. Gilbert , M. Shaw , A. Siddiqi , and M. S. Goodman , “Achieving the Health Equity Agenda Through Transformative Community‐Engaged Strategies,” Preventing Chronic Disease 20 (2023): E99, 10.5888/pcd20.230077. 37943729 PMC10684278

[35] K. Key , D. Furr‐Holden , E. Y. Lewis , et al., “The Continuum of Community Engagement in Research: A Roadmap for Understanding and Assessing Progress,” Progress in Community Health Partnerships 13, no. 4 (2019): 427–434, 10.1353/cpr.2019.0064. 31866597

[36] R. K. Chandler , J. Villani , T. Clarke , E. F. McCance‐Katz , and N. D. Volkow , “Addressing Opioid Overdose Deaths: The Vision for the Healing Communities Study,” Drug and Alcohol Dependence 217 (2020): 108329, 10.1016/j.drugalcdep.2020.108329. 33075691 PMC7528974

